# Prevalence and aggressiveness of papillary thyroid carcinoma in surgically-treated graves’ disease patients: a retrospective matched cohort study

**DOI:** 10.1186/s40463-019-0364-5

**Published:** 2019-08-28

**Authors:** Eunice You, Marco A. Mascarella, Abrar Al Jassim, Veronique-Isabelle Forest, Michael P. Hier, Michael Tamilia, Marc Pusztaszeri, Richard J. Payne

**Affiliations:** 1 McGill University, Montreal, Quebec Canada; 2 McGill Department of Otolaryngology – Head and Neck Surgery, Jewish General Hospital, Montreal, Quebec Canada; 3 McGill Department of Epidemiology, Biostatistics and Occupational Health, Montreal, Quebec Canada; 4 Western Otolaryngology – Head and Neck Surgery, London, Ontario Canada; 5 Division of Endocrinology, Jewish General Hospital, Montreal, Quebec Canada; 6 Department of Pathology, Jewish General Hospital, Montreal, Quebec Canada

**Keywords:** Graves’ disease, Papillary thyroid carcinoma, Aggressive carcinoma, Matched cohort, Outcomes

## Abstract

**Background:**

Reported rates of thyroid cancer in Graves’ disease (GD) vary widely. The aim of this study was to evaluate the prevalence of papillary thyroid carcinoma (PTC), including aggressive forms, in GD compared to matched controls undergoing thyroidectomy. Furthermore, it seeks to elucidate any patient- or tumour-associated factors predictive of malignancy or an aggressive course.

**Methods:**

We performed a matched cohort study of GD patients undergoing thyroidectomy at our institution between 2006 to 2018. Clinicodemographic factors, preoperative characteristics, surgical factors, final histopathology as well postoperative course were collected. Aggressive PTC was defined as evidence of lymph node metastasis, extrathyroidal extension, gross vascular invasion and/or aggressive histologic variants. Prevalence of PTC was compared with sex, age and nodule size-matched euthyroid patients that underwent thyroidectomy in the same time period.

**Results:**

A total of 132 patients were included in the study with a mean age of 46 (±14) years. Malignancy was identified in 36/66 (55%) patients with GD; 20/66 (30%) were incidental carcinomas and 9/66 (14%) were associated with aggressive pathologic features. In the aggressive group, lymph node metastasis to the central compartment was present in 8 (12%) cases, extrathyroidal extension in 4 (6%) cases and one (1.5%) patient had a diffuse sclerosing tumor variant. No significant differences in outcome were found between the two groups. GD patients were more likely to have incidental carcinomas (*p* = 0.035). Adjusting for baseline patient characteristics, GD patients demonstrated an increased likelihood of harbouring a malignancy (odds ratio (OR) = 2.67; 95% confidence interval (CI) 1.00–7.18) compared to controls.

**Conclusion:**

More than half of patients with GD undergoing thyroidectomy had concurrent thyroid malignancy with aggressive features present in 14% of patients. GD may confer a heightened risk of thyroid cancer; thyroid nodules should therefore be carefully investigated.

## Introduction

Graves’ disease (GD), also known as toxic diffuse goiter, is the most common cause of hyperthyroidism [1]. Such patients are thought to have a heightened risk of thyroid cancer but prevalence varies widely, with rates of up to 21.1% being reported in GD patients [2]. A collective review by Belfiore et al. observed that the malignancy rate of palpable thyroid nodules in GD patients ranged from as low as 2.3% to up to 45.8% [3] while the malignancy rate of palpable nodules occurring in the general population hovered around 5% [4, 5]. However, many of the included studies did not account for incidental carcinomas. Furthermore, preoperative features were limited and therefore, the malignancy rate in clinically “suspicious” nodules remains unclear.

The debate is further complicated by conflicting studies on the biological behaviour of thyroid cancers in patients with concurrent GD. Some authors have identified a more aggressive course in the GD population, with increased frequency of invasion and nodal metastasis [6–10]. In one study, the cumulative risk for metastasis was estimated be threefold higher in GD patients [11]. Higher rates of mortality [12, 13], regardless of small primary tumour size [9], were also reported. Given these findings, Pellegriti et al. recommended early diagnosis and more aggressive treatment involving a total thyroidectomy and lymph node dissection in all GD patients followed by radioactive iodine (I-131) therapy [6]. In contrast, other studies reported no significant difference in clinical outcomes between GD patients and sex- and age-matched controls [14, 15].

Given a lifetime risk of 3% in women and 0.5% in men for GD [16], ongoing epidemiologic data on the association between GD and cancer is needed to clarify these debates and better guide our goals of management in these patients. The aim of our study was therefore to study the prevalence of papillary thyroid carcinomas (PTC) in a cohort of GD patients treated with thyroidectomy, including incidental carcinomas and carcinomas associated with aggressive features, as well as to elucidate any factors predictive for malignancy or an aggressive course.

## Methods

### Study design

A multicentre retrospective matched cohort study was conducted at two tertiary care centers in Montreal, Canada. Consecutive patients with GD who underwent thyroidectomy from January 2006 to November 2018 were included in the study. The diagnosis of GD was made in all patients through a combination of clinical findings, imaging criteria on scintigraphy and laboratory findings of suppressed thyroid stimulating hormone, elevated free triiodothyronine or free thyroxine and/or positive thyroid-receptor antibody tests. Exclusion criteria included those with previous head and neck malignancy or history of neck radiation, those who did not have a formal diagnosis of GD by an otolaryngologist or endocrinologist or those who did not undergo surgical management. Indications for surgery included hyperthyroidism refractory to medication, refusal or intolerance to antithyroid drugs, presence of suspicious or malignant thyroid nodules by fine needle aspiration (FNA) biopsy, presence of large volume goiters resulting in compressive symptoms, Graves’ ophthalmopathy, or patient’s preference.

Preoperative work-up for included thyroid nodules included ultrasound imaging, thyroid uptake scans and/or FNA biopsies. Hypoechogenicity, microcalcifications, irregular borders and/or a predominantly solid nodule were considered to be suspicious features on ultrasound. FNA cytology was characterized following the Bethesda classification system. The McGill Nodule Thyroid Score (MNTS), a clinical scoring system estimating the risk of malignancy of thyroid nodules based on various clinical, laboratory, imaging and cytology parameters, was also calculated [17]. In the event that there were several FNA, the most worrisome sample (larger size, higher Bethesda score, etc.) was used for comparison with the final surgical pathology.

Patients typically underwent a total thyroidectomy for management of GD (medically refractory hyperthyroidism, large goiter, compressive symptoms, ophthalmopathy), or other indications including presence of highly suspicious nodules, positive lymph nodes identified pre-operatively and/or patient preference. However, both total and subtotal thyroidectomies were included in our analysis. Completion thyroidectomies were also performed if significant aggressive tumors or metastatic disease are identified on final pathological report. An elective sampling of central compartment neck lymph nodes with a sentinel lymph node biopsy using methylene blue is performed for all cases of suspected or confirmed malignancy.

A selected number of patients also underwent whole body scan radioactive iodine therapy (I-131) postoperatively, typically for various indications including large tumor sizes > 2 cm, presence of aggressive features, presence of aggressive histological variants or residual locoregional disease. Most GD patients were followed long-term in the endocrinology department after the surgery and the presence of regional or distant recurrence was evaluated at each visit.

The study was approved by the institutional review board of the McGill University Health Centre Research Ethics Board (REB) in Montreal, Quebec (2019–1532).

### Primary outcome

Our primary outcome was the presence of malignancy on final postoperative pathology reports. Additionally, histologic variants of PTC including aggressive types such as tall cell, columnar cell, solid/trabecular, diffuse sclerosing and hobnail were recorded. Pathologic features such as presence of extrathyroidal extension, lymph node metastasis and/or lymphatic or vascular invasion were also recorded.

Papillary thyroid microcarcinoma (PTmC) was defined as any PTC that was under 10 mm in diameter. Aggressive PTC was defined as any PTC associated with either lymph node metastasis, extrathyroidal extension, gross vascular invasion and/or aggressive histologic variants as previously mentioned. The term incidental carcinoma was used to designate all PTmC without any aggressive features.

### Covariates

The medical records of each patient were reviewed, and the data recorded in an electronic data collection form. We sought out all relevant information regarding sociodemographic factors (age, sex), surgical procedure (surgeon, date, surgical treatment, sentinel lymph node biopsy and central neck dissection), pre-operative characteristics (suspicious features on ultrasound, cold nodules on thyroid scans, MNTS, reason for surgery), final pathology (benign or malignant, size of the tumor, presence of pathologic features) and post-operative follow-up (I-131 therapy, date of last follow-up, recurrence and survival).

### Matched cohort

The final outcomes were compared with sex-, age- and nodule size-matched euthyroid control that underwent thyroidectomy in the same time period.

### Statistical analysis

Descriptive statistics was performed using non-parametric testing of Chi-squared analysis. McNemar’s test was used for categorical pairs and Wilcoxon’s signed-rank test was used for continuous measures. Subsequently, multiple logistic regression was used to ascertain the association of various preoperative characteristics to prevalence of malignancy. A *p*-value < 0.05 was considered to be statistically significant. All statistical analyses were performed using R software (version 3.4, Vienna).

## Results

During the study period, 66 GD patients underwent thyroidectomy, of which 54 (82%) were women. The average age of patients was 48 ± 14 years, range 16–78 years. A thyroid nodule was present in 51 (77%) of these patients: 18 (27%) had a solitary nodule and 33 (50%) had a multinodular thyroid. The average size of the dominant nodule, which was determined based on values recorded on preoperative ultrasounds, was 2.1 ± 1.3 cm. The average MNTS score was 5.0 ± 4.6. Of those with FNA biopsies, results were varied with Bethesda 1 (*n* = 9), Bethesda 2 (*n* = 15), Bethesda 3 (*n* = 15), Bethesda 5 (*n* = 2) and Bethesda 6 (*n* = 6) results reported.

Thirty-six patients (55%) had PTC as confirmed by postoperative histopathology of which 16 (44%) were > 10 mm in size. Multifocal disease was identified in 34 (52%) cases. Nine patients out of 66 (14%) had a PTC associated with aggressive features; lymph node metastasis to the central compartment was present in each of the 8 (12%) cases, extrathyroidal extension in 4 (6%) cases and one case (1.5%) of diffuse sclerosing histologic variant was reported. Gross vascular invasion was not identified in any cases. Average tumor size, which was determined based on final pathology reports, was 2.3 ± 1.3 cm.

When compared to an age, sex and nodule size-matched cohort, we found that both MNTS and Bethesda category were significantly higher in the control group (*p* = 0.03 and *p* = 0.04 respectively). Despite this, a higher incidence of malignancy was observed in the GD cohort although these cancers were significantly more likely to be incidental carcinomas (*p* = 0.04). Furthermore, upon adjusting for the baseline MNTS and FNA, which were higher in the control group, GD patients demonstrated a higher likelihood of malignancy (odds ratio (OR) = 2.67; 95% confidence interval (CI) 1.00–7.18) and of having an aggressive cancer (OR = 1.48; 95% CI 0.45–4.90), although the trend did not reach significance.

Another finding of note was the elevated prevalence of nodular GD of 77% (51/66), which is significantly higher than most other reports which hover around 30% [1, 18–20]. The risk of carcinoma in nodular GD was found to be 40% (20/51) which is in agreement with previous studies that show thyroid nodules among GD harbours a 42% risk of PTC [21]. We also calculated the rate of malignancy in those with no concerning features (*n* = 45) compared to those with nodules that were preoperatively labeled as “suspicious” (*n* = 21). The malignancy rates (excluding non-incidental carcinomas) were 8/45 (17%) and 8/21 (38%) respectively. Lastly, in the 16 patients with confirmed non-incidental PTC, half had been identified as suspicious nodules, with a higher average MNTS score of 7.71 ± 5.97 and more nodules with Bethesda Category 5/6 than controls (*p* = 0.00148 and *p* < 0.00001 respectively.)

Radioactive iodine ablation therapy was performed in 9 (14%) patients with complete response. The average length of follow-up for the Graves’ disease patients was 48 ± 34 months. Regional or distant recurrence was not reported in patients in either groups and an overall survival rate of 98% was observed, with only one death reported several years later from an unrelated glioblastoma (Table 1).

**Table 1:** Clinicopathological characteristics of the study population

Variables	GD Group	Control Group	*p-*value
Age (years)	46 ± 14 (16–78)	46 ± 14 (19–75)	0.236
Gender			
Female	54 (82%)	54 (82%)	1.000
Male	12 (18%)	12 (18%)	1.000
MNTS	4.92 ± 4.6	8.63 ± 5.7	*0.03
Bethesda Category	2.70 ± 1.5	3.82 ± 1.5	*0.04
Nodule status			
Without nodule	15 (23%)	17 (26%)	0.788
With nodule			
Solitary nodule	18 (27%)	26 (39%)	0.286
Multinodular	33 (50%)	23 (35%)	
Nodule (cm)	2.1 ± 1.3 (0.4–6.0)	2.2 ± 1.2 (0.5–5.5)	0.524
Pathology			
Benign	30 (45%)	35 (53%)	0.385
If malignant	36 (55%)	31 (47%)	
Incidental carcinoma	20 (31%)	9 (14%)	*0.04
Papillary carcinoma	7 (11%)	9 (13%)	0.06
Aggressive	9 (13%)	13 (20%)	0.55
Mean tumor size (cm)	2.3 ± 1.3	1.8 ± 0.5	*0.05
Lymph node metastasis	8 (12%)	6 (9%)	0.599
Extrathyroidal extension	4 (6%)	5 (7.5%)	1.00
Invasion			
Lymphatic	4 (6%)	0 (0%)	N/ANéA
Vascular	0 (0%)	0 (0%)	N/A
Radioactive iodine therapy			
Yes	9 (14%)	9 (14%)	1.000
No	57 (86%)	57 (86%)	
Recurrence			
Yes	0 (0%)	0 (0%)	1.000
No	66 (100%)	66 (100%)	
Survival on latest follow-up			
Yes	65 (98%)	66 (100%)	1.000
No	1 (2%)	0 (0%)	

Data are presented as mean ± SD (range) or mean (percentage)

FNA = fine needle aspiration; MNTS = McGill Nodule Thyroid Score

*p < 0.05

Preoperative characteristics of the GD group were also extracted. Indications for surgery were varied and included airway compromise in 2 (3%) patients, large goiters with compressive symptoms in 15 (23%), uncontrolled hyperthyroidism in 14 (21%), Graves’ ophthalmopathy in 15 (22%) and patient preference in 7(11%). In comparison, all patients in the matched cohort group underwent surgery for suspicion of cancer. Furthermore, twenty-one (32%) patients had concurrent nodules with suspicious features on pre-operative work-up, either on imaging, estimated clinical risk score or FNA biopsy results. A thyroid scan was done in 39 (59%) patients and revealed cold nodules in 25 (38%) patients. Ultrasound was performed in 47 (71%) patients and revealed suspicious features including hypoechogenicity, increased vascularity, microcalcifications, irregular borders and dominant solid component in 27 (41%) nodules (Table 2).

**Table 2:** Preoperative characteristics of GD Group

Features	Number in GD Group
Indications for surgery	
Airway compromise	2 (3%)
Large goiter with compressive symptoms	15 (23%)
Medication refractory hyperthyroidism	14 (21%)
Graves’ ophthalmopathy	15 (22%)
Concurrent suspicion for cancer	21 (32%)
Patient preference	11 (17%)
Thyroid uptake scan (*n* = 39)	
Cold nodules	25 (64%)
No nodules or diffuse uptake	14 (36%)
Ultrasound (*n* = 47)	
With suspicious features	25 (53%)
Hypoechogenicity	11 (23%)
Microcalcifications	11 (23%)
Irregular borders	2 (4%)
Solid dominant	5 (11%)
With benign features	22 (47%)

Results are presented as number (percentage)

There were no significant differences in clinicodemographic features between patients with benign and malignant disease (Table 3.) On univariate analysis, we did not identify any clinicodemographic features (sex, age, preoperative nodule size and thyroid size) associated with an increased likelihood of malignancy or having an aggressive course with *p* < 0.05.

**Table 3:** Comparison of clinicodemographic features between patients with benign disease versus malignant outcomes

	Benign (*n* = 30)		Malignancy (*n* = 36)		*p value* (Chi-square)
	No.	%	No.	%	
Age (years)					
≤ 45	14	40	17	47	0.96
> 45	16	60	19	53	
Sex					
Male	5	17	7	20	0.77
Female	25	83	29	80	
Nodule (cm)					
≤ 2.0	12	55	20	71	0.21
> 2.0	10	45	8	29	
Thyroid (g)					
≤ 30	12	40	19	52	0.30
> 30	18	60	17	48	

## Discussion

Graves’ disease is found in about 0.5% of the population and makes up to 80% of cases of hyperthyroidism [22]. Reports on the association between GD and cancer remain inconsistent. The large discrepancy between individual studies may be due to non-comparable parameters of prognosis and outcomes, varying indications for surgery, extent of histological examination, genetic background and even geographical differences.

Our retrospective cohort study shows a malignancy rate of 36/66 (55%) among GD patients undergoing surgery. Excluding microcarcinomas, which are generally considered incidental findings with good prognosis, we are left with a prevalence of PTC in 16/66 (24%). These are consistent with the rates of 2.3 to 45.8% (mean 16.9%) that have previously been reported in the literature [23]. That being said, the prevalence of aggressive types of thyroid cancer among GD patients is not well defined in the literature. In this study, we defined any PTC associated with lymph node metastasis, extrathyroidal extension, gross vascular invasion and/or histologic variants known to be aggressive as aggressive carcinomas and found a prevalence of 9/66 (14%). We identified one case of diffuse sclerosing variant. On the whole, the clinical course was optimistic for both the GD cohort and the control group with no regional or distant recurrence noted in either groups at the latest follow-up.

We noted a higher baseline MNTS score and Bethesda score in the non-GD patients, likely as a significant number of GD patients were operated for control of GD rather than suspicion for cancer. However, upon adjusting for these features on multiple logistic regression, GD appeared to be associated with a higher likelihood of cancer as well as aggressiveness, although the trend did not reach significance for the latter.

Overall, this study demonstrates a high prevalence of thyroid carcinoma in GD patients, and identifies a proportion of these having aggressive features. As such, clinical concern for concurrent thyroid cancer in the initial evaluation of patients with GD, particularly those with suspicious nodules may confer an increased risk of malignancy [1].

Although we did not identify any clinicodemographic features associated with increased likelihood of malignancy or aggressive course, various epidemiological risk factors have been well established in the literature. There is a well-documented gender disparity in papillary thyroid cancer, with a 3-fold increase in incidence in females but a lower disease-free survival and higher mortality observed in males [24–26]. Age is also a key prognostic indicator for more aggressive cancers, with increased likelihood of having histologic variants and greater mortality [27]. Meanwhile nodularity has also been investigated for its impact on thyroid cancer, with possible increased risk of malignancy identified in nodules greater than 2 cm [28].

Despite these findings, the current study has several limiting factors. It is difficult to obtain an entire GD cohort as only those undergoing surgery were included, resulting in an inherent selection bias although consecutive patients were included. The study covers a long period for which patient selection criteria, extent of thyroidectomy and accuracy of histopathological analyses may have changed significantly. We also chose to focus on the disease characteristics such as nodule, tumour or thyroid sizes rather than patient characteristics such as family history of cancer, exposure to ionizing radiation, smoking history which were not easily available for each patient but would be worth studying. Clinical parameters such as thyroid hormone levels or thyroid antibodies were excluded since many patients had been well-controlled with medication prior to being referred to our centre. We also did not identify any clinicodemographic features such as sex, age, preoperative nodule size and thyroid size that were significantly associated with an increased likelihood of malignancy or having an aggressive course. However, we did not expect to detect differences as our sample size was limited. Performing a study with a larger health database and obtaining more complete pre-operative and post-operative data would be the next step if the study were to be pursued further.

## Conclusion

There is a higher prevalence of PTC in GD patients undergoing surgery, and that PTC with aggressive histopathologic features represents a significant proportion of cases. Furthermore, having GD may confer an increased likelihood of cancer. As a result, patients with GD who have thyroid nodules should be carefully investigated.

## Data Availability

The datasets supporting the conclusions of this article are included within the article.

## Abbreviations

CI: Confidence interval
GD: Graves’ Disease
OR: Odds ratio
PTC: Papillary thyroid carcinoma
PTmC: Papillary thyroid microcarcinoma

## References

[1] Gerenova J, Buysschaert M, De Burbure C, Daumerie C (2003). Prevalence of thyroid cancer in graves’ disease: a retrospective study of a cohort of 103 patients treated surgically. Eur J Intern Med.

[2] Pazaitou-Panayiotou K, Michalakis K, Paschke R (2012). Thyroid cancer in patients with hyperthyroidism. Horm Metab Res.

[3] Belfiore A, Russo D, Vigneri R, Filetti S (2001). Graves' disease, thyroid nodules and thyroid cancer. Clin Endocrinol.

[4] Belfiore A, La Rosa GL, La Porta GA, Giuffrida D, Milazzo G, Lupo L (1992). Cancer risk in patients with cold thyroid nodules: relevance of iodine intake, sex, age, and multinodularity. Am J Med.

[5] Mazzaferri EL (1993). Management of a solitary thyroid nodule. N Engl J Med.

[6] Pellegriti G, Belfiore A, Giuffrida D, Lupo L, Vigneri R (1998). Outcome of differentiated thyroid cancer in Graves' patients. J Clin Endocrinol Metab.

[7] Belfiore A, Garofalo MR, Giuffrida D, Runello F, Filetti S, Fiumara A (1990). Increased aggressiveness of thyroid cancer in patients with Graves' disease. J Clin Endocrinol Metab.

[8] Boutzios G, Vasileiadis I, Zapanti E, Charitoudis G, Karakostas E, Ieromonachou P (2014). Higher incidence of tall cell variant of papillary thyroid carcinoma in Graves' disease. Thyroid..

[9] Ozaki O, Ito K, Kobayashi K, Toshima K, Iwasaki H, Yashiro T (1990). Thyroid carcinoma in Graves' disease. World J Surg.

[10] Mazzaferri EL (1990). Thyroid cancer and Graves' disease. J Clin Endocrinol Metab.

[11] Phitayakorn R, McHenry CR (2008). Incidental thyroid carcinoma in patients with graves’ disease. Am J Surg.

[12] Pazaitou-Panayiotou K, Perros P, Boudina M, Siardos G, Drimonitis A, Patakiouta F (2008). Mortality from thyroid cancer in patients with hyperthyroidism: the Theagenion Cancer hospital experience. Eur J Endocrinol.

[13] Hayes FJ, Sheahan K, Heffernan A, McKenna TJ (1996). Aggressive thyroid cancer associated with toxic nodular goitre. Eur J Endocrinol.

[14] Chao T-C, Lin J-D, Jeng L-B, Chen M-F (1999). Thyroid cancer with concurrent hyperthyroidism. Arch Surg.

[15] Yano Y, Shibuya H, Kitagawa W, Nagahama M, Sugino K, Ito K (2007). Recent outcome of Graves' disease patients with papillary thyroid cancer. Eur J Endocrinol.

[16] Pokhrel B, Bhusal K (2017). Graves disease.

[17] Sands NB, Karls S, Amir A, Tamilia M, Gologan O, Rochon L, et al. McGill Thyroid Nodule Score (MTNS): "rating the risk," a novel predictive scheme for cancer risk determination. Journal of otolaryngology - head & neck surgery = Le Journal d'oto-rhino-laryngologie et de chirurgie cervico-faciale. 2011;40 Suppl 1:S1–13. 21453655

[18] Tam AA, Kaya C, Kılıç FBM, Ersoy R, Çakır B (2014). Thyroid nodules and thyroid cancer in graves’ disease. Arq Bras Endocrinol Metabol.

[19] Erbil Y, Barbaros U, Özbey N, Kapran Y, Tükenmez M, Bozbora A (2008). Graves' disease, with and without nodules, and the risk of thyroid carcinoma. J Laryngol Otol.

[20] Mishra A, Mishra SK. Thyroid nodules in Graves' disease: implications in an endemically iodine deficient area. 2001. 11832639

[21] Weber KJ, Solorzano CC, Lee JK, Gaffud MJ, Prinz RA (2006). Thyroidectomy remains an effective treatment option for graves’ disease. Am J Surg.

[22] Chen Y-K, Lin C-L, Chang Y-J, Cheng FT-F, Peng C-L, Sung F-C (2013). Cancer risk in patients with Graves' disease: a nationwide cohort study. Thyroid..

[23] Menon R, Nair CG, Babu M, Jacob P, Krishna GP. The outcome of papillary thyroid Cancer associated with graves’ disease: a case control study. J Thyroid Res. 2018;2018. 10.1155/2018/8253094 PMC5964588 29854383

[24] Rahbari R, Zhang L, Kebebew E (2010). Thyroid cancer gender disparity. Future Oncol.

[25] Cady B, Rossi R (1988). An expanded view of risk-group definition in differentiated thyroid carcinoma. Surgery..

[26] Grubbs EG, Rich TA, Li G, Sturgis EM, Younes MN, Myers JN (2008). Recent advances in thyroid cancer. Curr Probl Surg.

[27] Kwong N, Medici M, Angell TE, Liu X, Marqusee E, Cibas ES (2015). The influence of patient age on thyroid nodule formation, multinodularity, and thyroid Cancer risk. J Clin Endocrinol Metab.

[28] Kamran SC, Marqusee E, Kim MI, Frates MC, Ritner J, Peters H (2013). Thyroid nodule size and prediction of cancer. J Clin Endocrinol Metab.

